# Exploring the role of Transcranial magnetic stimulation in cognitive impairment and sarcopenia: a narrative review

**DOI:** 10.3389/fnhum.2025.1601651

**Published:** 2025-07-03

**Authors:** Yuhong Wang, Weichao Li, Tingting Dong, Rui Xu, Qing Ai, Lili Yang, Xuesong Gai, Li Li

**Affiliations:** ^1^Department of Rehabilitation Medicine, The First People’s Hospital of Yunnan Province, Affiliated Hospital of Kunming University of Science and Technology, Kunming, Yunnan, China; ^2^Department of Orthopedics, The First People’s Hospital of Yunnan Province, Affiliated Hospital of Kunming University of Science and Technology, Kunming, Yunnan, China; ^3^Department of Emergency Trauma Surgery, The First People’s Hospital of Yunnan Province, Affiliated Hospital of Kunming University of Science and Technology, Kunming, Yunnan, China

**Keywords:** sarcopenia, cognitive impairment, transcranial magnetic stimulation, muscle-brain axis, oxidative stress, chronic inflammation

## Abstract

Sarcopenia and cognitive impairment (CI) are major contributors to disability among the elderly, diminishing their quality of life and placing also a significant burden on healthcare systems and societies. There are currently no effective treatments for sarcopenia and CI. Interestingly, recent research has revealed a strong connection between these two conditions. Although the exact mechanisms of this relationship remain unclear, factors such as chronic inflammation, mitochondrial oxidative stress, and impaired communication through the muscle-brain axis have been put forward. Transcranial magnetic stimulation (TMS) is a non-invasive brain stimulation technique that can modulate neural plasticity and reduce inflammation and oxidative stress, demonstrating considerable potential for improving CI and motor function disorders. In this review, we summarize the relationship between sarcopenia and CI and discuss their shared mechanisms of action. In addition, we aim to provide new research insights and treatment directions by describing the physiopathological mechanisms modulated by TMS and its therapeutic potential for treating CI and sarcopenia.

## 1 Introduction

As the aging population continues to grow, age-related medical conditions such as cognitive impairment (CI) and sarcopenia are increasingly becoming significant public health issues worldwide. Common cognitive disorders encountered in clinical practice include mild CI (MCI) and Alzheimer’s disease (AD). MCI refers to a condition where individuals experience mild impairments in memory, thinking, language, and other cognitive functions, but these do not result in significant challenges in daily living activities. As people age, the prevalence of MCI rises from year to year: among individuals aged 70–74, the prevalence of MCI is 10%, rising to as high as 25% in those aged 80–84 (Hansson et al., 2006; Petersen et al., 2018). MCI is often considered a preclinical stage of AD, with over 40% of MCI patients progressing to AD within 4–6 years (Hansson et al., 2006). AD is characterized by progressive cognitive and memory impairments, as well as a decline in the ability to perform daily activities. AD not only diminishes quality of life in those affected, but places also a significant burden on families and society. According to statistics, in 2022 the treatment costs for AD patients in the United States reached $321 billion, and it is projected that by 2050, these expenses will exceed $1 trillion (Wong, 2020). However, there are currently no effective therapies to cure or reverse MCI and AD. Current treatment methods mainly include pharmacotherapy, cognitive training, and lifestyle interventions. Although these treatments can delay to some extent disease progression, they still face various challenges, including adverse drug reactions, high economic costs, and lack of personalization. Further research and innovation are needed to address these issues and explore new patient-tailored treatment methods.

Sarcopenia is an age-related disease characterized by the progressive loss of skeletal muscle mass and function. Sarcopenia can lead to functional decline, disability, weakness, and falls, significantly impacting the quality of life and life expectancy of older adults. It is estimated that the disease affects at least 10%–16% of the global elderly population (Yuan and Larsson, 2023). Although the prevention and treatment of sarcopenia has become a focal point in global geriatric medicine research, there are currently no approved medications for its treatment. While testosterone and other anabolic steroids can improve muscle strength and quality to some extent, their side effects are significant and can lead to severe complications, such as prostate cancer in men and masculinization in women (Shin et al., 2018). Non-pharmacological treatments primarily involving resistance training and nutritional support can also improve sarcopenia; however, their effectiveness is limited, and the intervention period is relatively long, requiring at least three months or more (Cho et al., 2022). Moreover, physical activity among older adults decreases with age. Therefore, there is a pressing need to explore new intervention strategies to address sarcopenia. In recent years, a correlation between sarcopenia and CI has become evident. Sarcopenia increases the risk of CI, which can in turn further reduce muscle strength (Hu et al., 2022; Xing et al., 2023). Sarcopenia and CI are bidirectionally associated, and the potential common pathological mechanisms between the two may be related to factors such as chronic inflammation, mitochondrial oxidative stress, and deficits in the muscle-brain axis. Given the shared influencing factors, it is urgent to explore new approaches to delay the progression of both conditions.

Transcranial magnetic stimulation is a non-invasive brain stimulation technique that induces neuronal activity in specific areas of the cerebral cortex by applying brief magnetic field pulses to the scalp (Jannati et al., 2023). TMS is easy to operate, highly secure, and cost-effective, allowing for personalized non-invasive neural modulation that addresses many treatment shortcomings. Currently, TMS is widely used in the treatment of various mental and neurological disorders, including depression, spinal cord injuries, and chronic pain, particularly in patients for whom medication has proven ineffective (Tan et al., 2024). In addition, TMS can enhance neural plasticity and improve the functionality of neural networks (Wu et al., 2022; Han et al., 2023b), alleviate chronic inflammation and oxidative stress, and promote the secretion of neurotrophic factors, showing great potential in the improvement of cognitive and motor functions. With the goal of providing new ideas and directions for effective treatment, this review will discuss the relationship and common mechanisms between CI and sarcopenia, as well as the fundamental principles of TMS and its applications in these debilitating geriatric syndromes. We used the PubMed database for a selective literature search of papers published between January 2000 and January 2025. Our main search terms included “cognitive impairment,” “Alzheimer’s disease,” “mild cognitive impairment,” “sarcopenia,” “transcranial magnetic stimulation,” “repetitive transcranial magnetic stimulation,” “chronic inflammation,” “oxidative stress,” “the muscle-brain axis,” “motor dysfunction,” and “neural plasticity.” All the literature included in this review was written in English.

## 2 Association between sarcopenia and CI

There is a close relationship between sarcopenia and CI, two significant contributors to disability in the elderly. A cross-sectional study collected data on the aging population in China, Ghana, India, Mexico, Russia, and South Africa from 2007 to 2010, revealing a positive correlation between sarcopenia and MCI (Jacob et al., 2021). Further research by Hu et al. (2022) found that individuals with sarcopenia are 1.72 times more likely to develop MCI compared to those without the disease. Beeri et al. (2021) conducted a follow-up study over 5.6 years involving 1,175 elderly individuals. They found that patients with sarcopenia had an increased risk of developing AD and MCI, as well as a more rapid decline in cognitive abilities, independent of factors such as age, gender, education level, ethnicity, and height. Sarcopenia accelerates CI, increasing the risk of cognitive disorders in the elderly by 80% (Ramoo et al., 2022). However, it is currently unclear which specific indicators of sarcopenia affect CI. A study that investigated the components of sarcopenia and their impact on cognitive function found that grip strength and gait speed independently predicted psychomotor function and overall cognition, while muscle mass had a lesser effect on cognitive function (Sui et al., 2020). The decline in muscle function appears to be a driving factor behind CI, and early screening of gait speed and grip strength in the elderly may serve as an effective means of predicting cognitive decline (Chou et al., 2019; Peng et al., 2023).

However, CI may also affect sarcopenia. Research has found that the prevalence of sarcopenia among patients with CI is significantly higher (26.4%) than in individuals without CI (8.3%) (Pacifico et al., 2020). CI can lead to a decrease in motor coordination and physical activity, which not only accelerates muscle loss but may also result in metabolic abnormalities and further deterioration of bone health (Xing et al., 2023). Some studies suggest that CI may occur prior to motor impairments, indicating that elderly individuals with attention and executive function deficits tend to exhibit poorer motor performance and are more prone to mobility issues (Inzitari et al., 2007). Similarly, in a 6 years follow-up study of 1,793 elderly women, Atkinson et al. (2010) found that cognitive decline preceded reductions in grip strength and gait speed. The decline in cognitive function leads to decreased physical activity among older adults, resulting in diminished exercise performance. Over time, this contributes to a decline in muscle function and an increased risk of sarcopenia. Therefore, CI may be a risk factor for sarcopenia (Ohta et al., 2019; Cipolli et al., 2023). A recent meta-analysis has shown that MCI can lead to slower walking speeds in older adults. When MCI patients perform either single or dual tasks, their walking speed is reduced compared to healthy control groups, which may be related to the high cognitive load involved in task execution (Bahureksa et al., 2017). Furthermore, MCI can also affect static balance, leading to increased postural sway in patients while standing, which subsequently raises the risk of falls (Bahureksa et al., 2017). This evidence indicates that sarcopenia and CI mutually influence each other. Without effective intervention, this may lead to a vicious cycle, exacerbating the disability of the elderly.

## 3 Possible common pathological mechanisms of sarcopenia and CI

**FIGURE 1:**
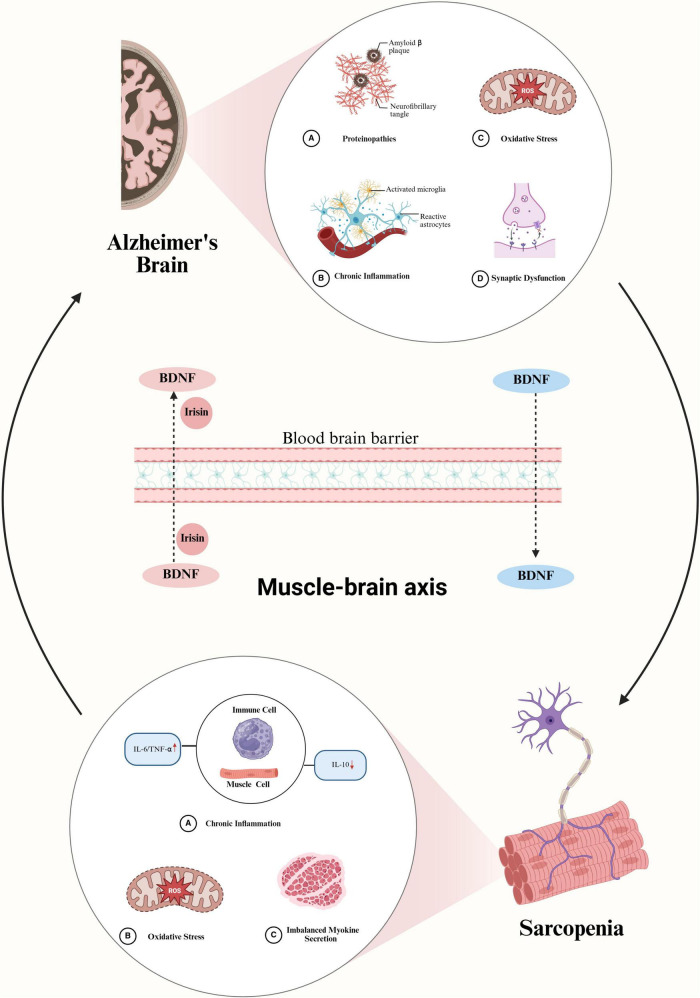
Possible mechanisms of interaction between Alzheimer’s Disease (AD)-related cognitive impairment (CI) and sarcopenia. In the brain of AD, amyloid-beta (Aβ) protein deposition activates astrocytes and microglia, leading to increased secretion of inflammatory factors, exacerbated oxidative stress, and synaptic dysfunction. These pathological changes reduce the amount of BDNF entering the circulatory system, which may induce sarcopenia. The occurrence of sarcopenia, in turn, exacerbates oxidative stress and chronic inflammation, prompting immune cells and muscle cells to secrete more inflammatory factors, accelerating muscle atrophy, and reducing the secretion of myokines such as BDNF and irisin. These changes further decrease the levels of BDNF and irisin entering brain tissue, exacerbating the pathological progression of AD and creating a vicious cycle of interaction between sarcopenia and cognitive impairment. BDNF, brain-derived neurotrophic factor; IL-6, interleukin 6; TNF-α, tumor necrosis factor alpha; IL-10, interleukin 10.

Sarcopenia and CI may share common pathophysiological mechanisms, with current focus placed on chronic inflammation, mitochondrial oxidative stress, and alterations in the muscle-brain axis (Figure 1).

### 3.1 Chronic inflammation

Aging is accompanied by a systemic inflammatory response, characterized by an increase in the secretion of pro-inflammatory cytokines such as interleukin-6 (IL-6), interleukin-1β (IL-1β), and tumor necrosis factor-alpha (TNF-α). This persistent inflammatory state leads to neuronal damage and death, ultimately resulting in various pathological manifestations (Wang C. et al., 2023). Chronic inflammation plays a significant role in the progression of CI. Compared to healthy subjects or patients with MCI, patients with AD exhibit significantly elevated concentrations of IL-6, IL-1β, and TNF-α in both plasma and cerebrospinal fluid (Swardfager et al., 2010; Culjak et al., 2020). Researchers have also clearly observed an increase in the expression of IL-6 and TNF-α in the brain tissue of AD mice (Yang et al., 2020; Shao et al., 2023). Concurrently, inflammatory pathways are activated, leading to an increase in the deposition of amyloid beta (Aβ) and Tau proteins. Inflammation exacerbates CI in mice, while reducing the expression of inflammatory factors can improve cognitive function to some extent (Yang et al., 2020; Shao et al., 2023). Chronic inflammation is also an important factor affecting sarcopenia. Under normal physiological conditions, pro-inflammatory and anti-inflammatory cells within the body antagonize each other, maintaining a balance between the synthesis and degradation of skeletal muscle. When muscle atrophy occurs, the expression of pro-inflammatory cytokines increases, leading to enhanced degradation of skeletal muscle and inhibition of skeletal muscle protein synthesis, thereby impairing muscle function (Sharma and Dabur, 2020; Pan et al., 2021). Skeletal muscle dysfunction may further exacerbate chronic inflammation, creating a vicious cycle that further impairs muscle function. Higher levels of circulating inflammatory markers are significantly associated with lower skeletal muscle strength and muscle mass (Tuttle et al., 2020). Numerous studies have found that pro-inflammatory factors such as IL-6, TNF-α, and C-reactive protein are significantly elevated in the serum of patients with sarcopenia, while the anti-inflammatory factor IL-10 is notably decreased (Tuttle et al., 2020; Ying et al., 2022; Lin et al., 2024). At the same time, patients exhibit a decline in grip strength and lower limb muscle strength compared to healthy controls, and their physical activity and daily living activities also diminish accordingly. Nutritional support and exercise can reduce the expression of these inflammatory factors to some extent, and effectively improve the grip strength and skeletal muscle mass index in patients with sarcopenia (Chang et al., 2023). Additionally, certain methods that increase the expression of anti-inflammatory factors, such as IL-10, can alleviate muscle atrophy and delay sarcopenia (Zhang et al., 2024; Sun et al., 2025). Hence, both sarcopenia and CI demonstrate the detrimental effects of chronic inflammatory responses on cellular function, while also influencing each other, leading to a higher overall health risk for the elderly population.

### 3.2 Mitochondrial oxidative stress

Oxidative stress refers to the imbalance between oxidants and antioxidants in the body, leading to damage of lipids, proteins, and DNA (Dhapola et al., 2024). This type of damage can affect synaptic activity and neurotransmission, leading to neuronal death. Excessive accumulation of reactive oxygen species (ROS) within cells is a primary characteristic of oxidative stress. It is estimated that 90% of ROS in cells originate from mitochondria. Mitochondrial dysfunction exacerbates ROS production mainly through impaired oxidative phosphorylation reactions, which may trigger systemic oxidative stress (Balaban et al., 2005). Extensive research has found widespread mitochondrial dysfunction in AD. This disrupts intracellular calcium homeostasis, which leads to synaptic loss and neurodegeneration, ultimately affecting cognitive function (Elfawy and Das, 2019; Wang W. et al., 2020). Oxidative stress is closely related to the onset and progression of CIs. Nie et al. (2024) found that the levels of oxidative stress markers in the peripheral blood of AD patients were significantly elevated compared to healthy controls, while their antioxidant capacity was reduced. Studies also found significantly elevated levels of ROS in brain tissue of AD mice (Park et al., 2021; Du et al., 2022). Oxidative stress may affect cognitive function through several mechanisms. For instance, it may accelerate the aggregation of Aβ and the hyperphosphorylation of Tau protein, resulting in impaired learning and memory functions characteristic of AD and other neurodegenerative syndromes (Das and Ganesh, 2023). By damaging neurons, oxidative stress may trigger apoptosis and necrosis, leading to the loss of brain tissue (Chen et al., 2008). Additionally, oxidative stress triggers neuroinflammatory responses, leading to the activation of microglia and astrocytes, which further impairs neuronal function (Firdous et al., 2024). Notably, oxidative stress may also specifically lead to a reduction in cholinergic neurons; this affects the synthesis and release of neurotransmitters, resulting in synaptic dysfunction and consequently diminishing cognitive abilities (Martins et al., 2018; Liu Z. et al., 2022).

Mitochondria play a crucial role in energy supply and the maintenance of redox homeostasis in muscle cells. These organelles are the primary energy source for muscle fibers, and their dysfunction can lead to energy deficits that may prompt the death of motor neurons and muscle fibers (Alway et al., 2017). Moreover, a pernicious feedback loop of excessive accumulation of cellular pro-oxidants intensifies mitochondrial damage and degradation, exacerbating oxidative stress (Ferri et al., 2020). The latter is also an important factor affecting muscle function. During the aging process, the body’s antioxidant capacity decreases, leading to the accumulation of ROS and reactive nitrogen species (RNS) within muscle cells (Liguori et al., 2018). Compared to healthy subjects, patients with sarcopenia show a significant increase in oxidative stress markers such as malondialdehyde and 4-hydroxy-2,3-non-enal in plasma (Bellanti et al., 2018). These oxidative stress products promote muscle atrophy by increasing protein hydrolysis and reducing muscle protein synthesis, leading to decreased muscle strength and a reduction in muscle fiber count (Powers et al., 2011; Baumann et al., 2016). In addition, the accumulation of RNS/ROS can alter the morphology of the neuromuscular junction, leading to muscle denervation (Baumann et al., 2016). Furthermore, RNS/ROS were shown to reduce the release of acetylcholine in the synaptic cleft, thereby inhibiting the generation of muscle membrane action potentials (Baumann et al., 2016). At the same time, oxidative stress can also affect muscle excitation-contraction coupling and the cycling of cross-bridges within myofibrils, leading to a reduction in muscle mass and exacerbating the development of sarcopenia (Baumann et al., 2016; Liguori et al., 2018). In summary, both muscle and brain are highly sensitive to mitochondrial oxidative stress, which impairs signaling through the muscle-brain axis and compromises the body’s metabolic and neurophysiological functions.

### 3.3 The muscle-brain axis

The Muscle-Brain Axis refers to the bidirectional interactions and information transfer between skeletal muscle tissue and the brain via multiple physiological and biochemical pathways. Skeletal muscle is not only a main component of the locomotor system, but functions also as an endocrine organ by secreting numerous bioactive substances (myokines), including inflammatory factors (IL-6, IL-8, IL-15), brain-derived neurotrophic factor (BDNF), fibroblast growth factor 21, irisin, and extracellular vesicles such as exosomes (Li et al., 2025). These factors influence brain function through the circulatory system (Scisciola et al., 2021; Han et al., 2023a). Recent studies have revealed that exosomes secreted by skeletal muscle are capable of transporting proteins, nucleic acids, and other bioactive molecules across the blood–brain barrier, thus modulating the functions of brain cells (Han et al., 2023c). On the contrary, nerve cells also influence muscle metabolism and growth by secreting neurotransmitters and neurotrophic factors. As age progresses, the bidirectional regulatory interactions between skeletal muscle and the brain, collectively termed the muscle-brain axis, tend to diminish. This decline may predispose individuals to CI and sarcopenia. In this review, we primarily focus on the roles of BDNF and irisin. BDNF is widely expressed in the nervous system, and it affects neural function by influencing neurogenesis, neuronal growth and development, and synaptic plasticity (Gao et al., 2022). Researchers have detected a significant decrease in BDNF in the brain tissue (Peng et al., 2005), blood (Ng et al., 2019), and cerebrospinal fluid of AD patients (Forlenza et al., 2015). Insufficient BDNF can lead to reduced synaptic plasticity, thereby affecting learning ability and memory consolidation (de Pins et al., 2019). Research has found that increasing the expression of BDNF in brain tissue can promote the generation of hippocampal neurons, enhance dendritic spine density, and improve learning ability in AD mouse models (de Pins et al., 2019; Jiang et al., 2024). Deriving from both central and peripheral secretion sources, BDNF is also present in the circulatory system, influencing weight regulation and several other metabolic processes (Briana and Malamitsi-Puchner, 2018).

Several studies have indicated that neuronally-derived BDNF is capable of traversing the blood–brain barrier and entering the systemic circulation (Rasmussen et al., 2009). Following exercise, it has been observed that 70%–80% of the BDNF detected in circulation within 4 h is of cerebral origin (Di Raimondo et al., 2020). During exercise, muscle cells also secrete large amounts of BDNF through autocrine or paracrine mechanisms, which plays a crucial role in promoting muscle cell generation and differentiation, and motor neuron survival. Accordingly, increasing peripheral BDNF levels can enhance muscle strength, ameliorate exercise-induced skeletal muscle metabolism, and promote mitochondrial remodeling and recovery (Halievski et al., 2020; Chan et al., 2024). Furthermore, BDNF present in the peripheral circulation has the potential to traverse the blood–brain barrier, thereby influencing neuronal function and synaptic plasticity. For instance, research has shown that exosomes carrying BDNF in the peripheral circulation can effectively cross the blood–brain barrier in mouse models of Parkinson’s disease, offering substantial protection to dopaminergic neurons (Wang et al., 2024). However, the ability of exosomes to travel from the peripheral circulation to the brain remains a subject of ongoing debate within the academic community. It has been reported that exosomes injected peripherally are predominantly taken up by organs such as the liver and kidneys, with only a small fraction ultimately reaching brain tissue (Liu S. et al., 2022). Nonetheless, the neuroprotective benefits of exercise on the brain may, at least in part, be ascribed to BDNF synthesized by skeletal muscle (Nay et al., 2021).

Irisin is a myokine produced by muscle cells during exercise, primarily generated through the proteolytic cleavage of the membrane protein fibronectin type III domain-containing protein 5 (FNDC5). During exercise, muscle cells significantly upregulate the expression of FNDC5 and release large amounts of irisin into the circulation (Li et al., 2025). These irisin molecules can directly traverse the blood–brain barrier, thereby stimulating BDNF expression in the hippocampus and consequently enhancing cognitive function (Wrann et al., 2013; de Freitas et al., 2020). Irisin is intimately linked to the progression of AD. Research has shown that FNDC5/irisin levels are diminished in the hippocampus and cerebrospinal fluid of patients with AD, as well as in mouse models of AD (Lourenco et al., 2019). However, elevating FNDC5/irisin levels in the brain or peripheral blood can enhance synaptic plasticity and alleviate memory impairments in AD mouse models (Lourenco et al., 2019). Exercise is widely recognized as an effective strategy to delay the progression of AD. This beneficial effect may stem from the increased release of myokines, such as BDNF and irisin, which mediate the crosstalk between the brain and muscles, thereby slowing the progression of CI (Jaberi and Fahnestock, 2023). Moreover, irisin can reduce Aβ levels by enhancing the secretion of the Aβ-degrading enzyme neprilysin in astrocytes, an effect mediated through the inhibition of the IL-6/ERK-STAT3 signaling pathway (Kim et al., 2023). Irisin also plays a significant role in the development of sarcopenia. Studies have shown that individuals with sarcopenia have significantly lower levels of circulating irisin compared to healthy subjects (Zhang et al., 2025). Furthermore, circulating irisin levels are positively correlated with muscle mass. Similarly, the expression of FNDC5/irisin in the muscles of aging mice is significantly reduced. In cases where the FNDC5/irisin gene is knocked out, these mice exhibit an accelerated decline in muscle mass and strength. Conversely, augmenting the expression of recombinant irisin protein can effectively alleviate the progression of sarcopenia and mitigate aging-related metabolic disturbances (Guo et al., 2023).

Therefore, we speculate that CI leads to a decrease in BDNF levels in brain tissue, which in turn reduces the amount of BDNF entering the bloodstream, subsequently affecting muscle function and increasing the incidence of sarcopenia. In turn, when sarcopenia develops, muscle activity diminishes, leading to a reduction in the release of myokines, which correspondingly lowers BDNF and irisin levels in the brain. A vicious cycle is thereby established, which contributes to the exacerbation of CI (Figure 1).

## 4 Basic principles of TMS

Transcranial magnetic stimulation is based on Faraday’s law of electromagnetic induction. When a rapidly changing high-intensity electric current passes through a coil, it generates an instantaneous magnetic field around it. This changing magnetic field can penetrate the scalp and skull, influencing the activity of neurons in the cerebral cortex (Hallett, 2007; Rothwell, 2018). When the magnetic field strength reaches a sufficient level, the induced electric current can trigger the depolarization of neuronal populations in the target brain region, thereby producing neurophysiological effects (Halawa et al., 2021; Siebner et al., 2022).

Transcranial magnetic stimulation can be broadly categorized into single-pulse TMS (spTMS) and repetitive TMS (rTMS). spTMS consists of single-pulse discharges and is primarily used to detect neural activity in specific brain regions and assess the integrity of conduction pathways. When spTMS is applied to the primary motor cortex, it can activate the corticospinal tract and induce activity in the muscles innervated by the stimulated brain region (Williams et al., 2020; Matsumoto and Ugawa, 2024). Surface electromyography can effectively record the electrical activity of stimulated muscles, typically using the amplitude and latency of motor-evoked potentials (MEPs) as assessment indicators. The amplitude of MEPs reflects the excitability of the motor cortex, and changes in amplitude can indicate dysfunction in the cortical motor pathways. A decrease in MEP amplitude has been observed in various conditions, including multiple sclerosis (MS) and stroke (Vucic et al., 2023). The latency period of the MEP represents the conduction time of stimuli from the central nervous system to the periphery. It can be used to assess the integrity of the corticospinal tract by evaluating changes in central and peripheral conduction times (Wang Y. et al., 2023). In patients with spinal cord injuries, the latency of MEPs is prolonged to varying degrees, reflecting abnormalities in the corticospinal conduction pathways (Smith et al., 2000; Nakamae et al., 2010). rTMS modulates the excitability of target brain regions through repeated stimulation. When applied to the primary motor cortex, high-frequency (≥ 5 Hz) rTMS typically increases cortical excitability, while low-frequency (≤ 1 Hz) rTMS tends to decrease it (Hallett, 2007; Wang J. et al., 2020). Research has found that the therapeutic effects of a single session of rTMS stimulation can last approximately 30–60 min (Heide et al., 2006; Mendonça et al., 2021). However, after repeated treatments, rTMS produces cumulative effects that induce plastic changes in the brain. The mechanisms by which rTMS enhances brain plasticity may be related to modulation of long-term potentiation (LTP) and long-term depression (LTD) at synapses via multiple potential processes, including regulation of Ca^2+^ channels (Tan et al., 2013), activation of NMDA receptors (Qian et al., 2023), modulation of GABAergic interneurons and BDNF release (Hong et al., 2024). These mechanisms work in concert to regulate synaptic transmission efficiency, which in turn shapes downstream signaling pathways and ultimately drives changes in gene expression and synaptic structural remodeling. In this context, the rate and total amount of postsynaptic Ca^2+^ influx serve as critical determinants for the induction of either LTP or LTD (Huang et al., 2011). rTMS modulates synaptic strength by regulating Ca^2+^ influx into the postsynaptic membrane. Specifically, intermittent theta-burst stimulation (iTBS) induces a rapid and high-amplitude Ca^2+^ influx, which activates calcium/calmodulin-dependent protein kinase II (CaMKII) and protein kinase C, thereby triggering plasticity similar to LTP. Conversely, continuous theta-burst stimulation (cTBS) induces LTD-like plasticity by gradually increasing the total amount of Ca^2+^ influx (Huang et al., 2011; Suppa et al., 2022). Moreover, rTMS can also activate postsynaptic NMDA receptors, with the Ca^2+^ influx mediated by these receptors serving as a key trigger for synaptic plasticity (Suppa et al., 2022). Meanwhile, GABAergic interneurons play a significant role in synaptic plasticity by modulating inhibitory synaptic transmission. For example, iTBS enhances synaptic excitation by suppressing the activity of GABAergic interneurons, while cTBS inhibits synaptic excitation by enhancing the activity of GABAergic interneurons (Huang et al., 2011; Li et al., 2019). Furthermore, rTMS can also improve synaptic plasticity in the hippocampus by activating the BDNF/tropomyosin-related kinase B (TrkB) signaling pathway (Shang et al., 2019). LTP and LTD are involved in the regulation of learning and memory processes by modulating synaptic transmission, which is of significant importance in research addressing CI treatments.

In addition to enhancing brain plasticity, rTMS significantly reduces inflammation by modulating neuroimmune responses and cytokine expression. Specifically, rTMS promotes the transformation of microglia from the pro-inflammatory M1 phenotype to the anti-inflammatory M2 phenotype. This shift results in decreased release of pro-inflammatory cytokines, such as IL-6, IL-1β, and TNF-α, and increased secretion of anti-inflammatory cytokines, such as IL-10 and TGF-β (Luo et al., 2022; Zou et al., 2024). Moreover, rTMS modulates the polarization state of astrocytes, facilitating their transition from the pro-inflammatory A1 phenotype to the anti-inflammatory A2 phenotype, thereby effectively mitigating neuroinflammation (Sheng et al., 2023). Recent studies have further demonstrated that rTMS can increase the proportion of regulatory T cells (Tregs) in peripheral blood, enhancing immune regulatory functions and thereby suppressing neuroinflammation (Xie et al., 2024).

**FIGURE 2:**
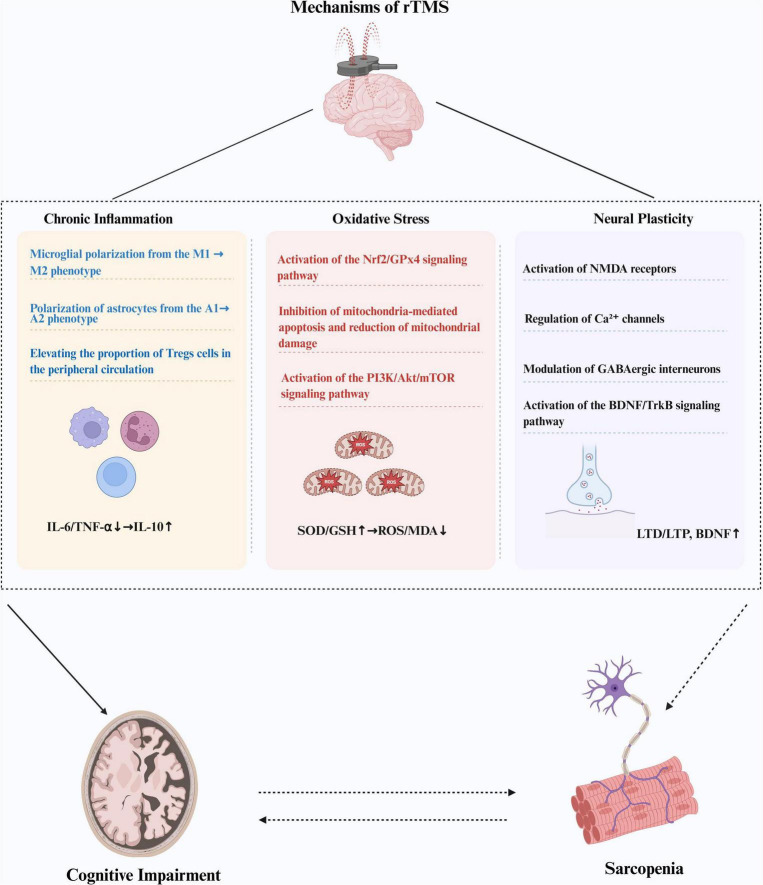
Repetitive TMS (RTMS) may exert potential therapeutic effects on CI and sarcopenia by alleviating chronic inflammation, mitigating mitochondrial oxidative stress, and enhancing neuroplasticity. While rTMS improves cognitive function, it may also positively influence sarcopenia; conversely, improvements in sarcopenia may facilitate the recovery of cognitive function. rTMS, repetitive transcranial magnetic stimulation; CI, cognitive impairment; IL-6, interleukin 6; TNF-α, tumor necrosis factor alpha; IL-10, interleukin 10; SOD, superoxide dismutase; GSH, glutathione; ROS, reactive oxygen species; MDA, malondialdehyde; LTD, long-term depression; LTP, long-term potentiation; BDNF, brain-derived neurotrophic factor.

Moreover, rTMS significantly mitigates oxidative stress through a multifaceted mechanism. First, rTMS activates the nuclear factor erythroid 2-related factor 2 (Nrf2)/glutathione peroxidase 4 (GPx4) signaling pathway, which in turn promotes the expression of key antioxidant enzymes, including GPx4 and superoxide dismutase (SOD) (Jin et al., 2024). This upregulation facilitates the efficient clearance of ROS within cells, resulting in a significant decrease in malondialdehyde (MDA) levels and thereby mitigating oxidative stress damage. Second, rTMS protects cells from oxidative stress by reducing mitochondrial damage, maintaining mitochondrial membrane integrity, and inhibiting mitochondria-mediated apoptosis pathways. Specifically, rTMS inhibits the activity of caspase-3 and caspase-9, reducing cell apoptosis and preserving neuronal integrity (Zong et al., 2020). Additionally, rTMS activates the phosphoinositide 3-kinase (PI3K)/Akt/mechanistic target of rapamycin (mTOR) signaling pathway (Stekic et al., 2022), which reduces inflammatory responses and oxidative stress, further enhancing its efficacy in improving oxidative stress. At the same time, rTMS has been found to enhance the proliferation of neural stem cells, thereby exerting a neuroprotective effect (Luo et al., 2024). Recent studies also found that rTMS can increase the conduction velocity of peripheral nerves controlling motor and sensory functions, alleviate muscle atrophy (Yan et al., 2023), and reduce neurogenic muscle damage (Peña-Toledo et al., 2021), demonstrating significant potential in improving motor function. Therefore, rTMS may be an effective treatment for CI and motor dysfunction (Figure 2).

## 5 Applications of TMS in the treatment of CI

Transcranial magnetic stimulation can serve as a diagnostic tool for CI, offering unique advantages in the non-invasive assessment of cortical excitability and plasticity. A recent meta-analysis confirmed the ability of TMS to detect a significant increase in cortical excitability, a decrease in cortical inhibition, and a reduction in cortical LTP-like plasticity in MCI and AD patients compared to healthy controls (Chou et al., 2022). Research has shown that patients with frontotemporal dementia exhibit increased cortical excitability, which is linked to impaired short-latency inhibition. Based on these characteristics, TMS can differentiate between various types of cognitive disorders (Antczak et al., 2021). In addition, clinical studies have shown that rTMS can effectively alleviate CI signs and symptoms. A randomized controlled trial showed that rTMS can enhance cognitive function in AD patients. The study employed functional magnetic resonance imaging (fMRI) to localize the left parietal region of the patients and utilized 20 Hz, 1,600-pulse rTMS for a 4 weeks intervention (Jung et al., 2024). The results showed that the AD Assessment Scale-Cognitive Subscale (ADAS-cog) scores of the patients were significantly reduced compared to the sham stimulation group. At the same time, the functional connectivity between the precuneus and hippocampus was enhanced, with these improvements persisting for at least 4 weeks following the end of treatment. Additionally, a meta-analysis by Chou et al. (2020) revealed variations in the therapeutic effects of rTMS applied at different frequencies in various brain regions. For instance, high-frequency (≥ 5 Hz) rTMS applied to the left dorsolateral prefrontal cortex (DLPFC) and low-frequency (≤ 1 HZ) rTMS on the right DLPFC primarily improve memory functions in patients with AD, while high-frequency rTMS on the right inferior frontal gyrus shows advantages in enhancing executive functions (Chou et al., 2020). The cerebellum is also an important target involved in the integration and regulation of cognitive networks. Patients with AD underwent a 4 weeks course of rTMS targeting the bilateral cerebellum. The stimulation frequency was set at 5 Hz, with an intensity of 90% of the resting motor threshold (RMT), delivering 2,000 pulses per session. Following the intervention, significant improvements were observed in the Mini-Mental State Examination (MMSE) scores (Yao et al., 2022). Additionally, notable enhancements were seen in overall cognitive function, episodic memory, executive function, language abilities, and visual-spatial skills. This change appears to be related to rTMS activating the cerebellar-thalamic-cortical circuit, enhancing functional connectivity in the brain. In addition, several studies have employed high-frequency rTMS at 20 Hz targeting the precuneus in patients with AD. The stimulation intensity was set at 100% of the RMT, with a total of 1,600 pulses delivered (Koch et al., 2018). The treatment consisted of ten sessions over 2 weeks (five sessions per week). Results showed that this stimulation significantly improved episodic memory but had limited effects on overall cognitive function. In the study by Koch et al. (2025), rTMS was applied to the precuneus of 32 patients with mild-to-moderate AD at the same frequency and intensity over a 52 weeks period. The results indicated that rTMS can slow cognitive decline, improve activities of daily living, and reduce behavioral disturbances. Therefore, intervention duration is a critical factor influencing therapeutic efficacy. Further research has compared the efficacy of single-target and multi-target stimulation, finding that the latter is more significant in improving cognitive function. Additionally, the effects of long-term treatment are superior to those of short-term treatment, with patients receiving more than 10 sessions showing a notable enhancement in cognitive function (Lin et al., 2019; Wang X. et al., 2020). In terms of frequency selection, 20 Hz rTMS was deemed more effective than 10 or 1 Hz (Wang X. et al., 2020).

Animal experiments have explored various potential mechanisms by which rTMS may improve CI. Wang et al. (2015) administered rTMS at 1, 10, and 15 Hz to 3xTg-AD mice over a 4 weeks period. The findings revealed that only 15 Hz rTMS significantly enhanced cognitive function, upregulated BDNF expression, strengthened hippocampal LTP, and decreased cortical excitability. Further research indicated that enhanced LTP may be related to increased synaptic density and structural changes induced by rTMS. Post-synaptic density protein 95 (PSD-95) and synaptophysin are expressed in pre-synaptic and post-synaptic sites, respectively, and serve as markers for synaptic density. Following rTMS, an increase in the expression of PSD-95 and synaptophysin proteins was observed in mouse brain tissue. Electron microscopy also revealed an increase in PSD thickness and a reduction in the width of synaptic clefts, suggesting that rTMS may increase synaptic density and structural plasticity (Ma et al., 2019). Interestingly, rTMS was reported to attenuate oxidative stress in mice with CI. RTMS exerts multifaceted neuroprotective effects in AD model mice by attenuating glutamate excitotoxicity via the PI3K/Akt/GLT-1 signaling pathway. Experiments revealed that 3 weeks of continuous rTMS treatment at 25 Hz and 80% of the device’s maximum output power significantly reduced Aβ deposition in the brain tissue of AD mice. Further mechanistic studies indicated that rTMS not only significantly downregulated the expression levels of pro-inflammatory cytokines IL-6 and TNF-α but also decreased the levels of ROS and malondialdehyde while increasing the levels of the antioxidants superoxide dismutase and glutathione. These results suggest that the regulation of the PI3K/Akt/GLT-1 signaling pathway may be a key mechanism by which rTMS ameliorates AD pathology (Cao et al., 2022). Ferroptosis, an iron-dependent form of programmed cell death, is also an important cause of CI. Excessive accumulation of iron ions within cells leads to mitochondrial dysfunction, which exacerbates oxidative stress and ultimately leads to ferroptosis (Reichert et al., 2020). Experiments in senescence-accelerated mouse prone 8 (SAMP8) mice, used to model aging-related CI, showed that high-frequency rTMS can alleviate ferroptosis in brain tissue by increasing the expression of Gpx4, system xc-, and Nrf2, thereby reducing oxidative stress and protecting neurons from damage (Xu et al., 2024). Additionally, reducing neuroinflammation can also alleviate CI. Research has shown that applying high-frequency (20 Hz) rTMS to AD mice for two consecutive weeks effectively reduced Aβ levels in brain tissue. Further investigations revealed that rTMS can decrease the expression of pro-inflammatory factors IL-6 and TNF-α and modulate the PI3K/Akt/NF-κB pathway to mitigate neuroinflammation (Li et al., 2021). Acetylcholine is an excitatory neurotransmitter that plays a crucial role in learning and memory. McNerney et al. (2022) found that rTMS upregulates the expression of the BDNF gene and the activity of acetylcholinesterase in AD mice, thereby reducing CI.

## 6 Explore the therapeutic potential of rTMS in sarcopenia

Early research suggested that magnetic stimulation holds great potential in improving muscle function, which suggested its utility to treat sarcopenia. Evidence provided by Stölting et al. (2016) indicates that magnetic stimulation can promote nerve and muscle regeneration by reducing inflammation, facilitating the aggregation of acetylcholine receptors, and inducing the maturation of neuromuscular junctions. Also, magnetic stimulation may prevent muscle atrophy in the lower limbs of stroke patients (Suzuki et al., 2020). Moreover, evidence suggests that rTMS can alleviate muscle atrophy related to neurodegenerative diseases. Peña-Toledo et al. (2021) reported significant muscle atrophy in guinea pigs with experimental autoimmune encephalomyelitis (EAE), a condition that mimics key aspects of MS, with the cross-sectional area of type 1 and type 2 fibers in the soleus muscle decreasing by 28% and 38%, respectively. Notably, following rTMS muscle atrophy was effectively reversed, while lipid peroxides were also significantly reduced. This suggested that rTMS may alleviate muscle atrophy by inhibiting oxidative stress. Indeed, a clinical study showed that rTMS can enhance muscle strength in patients with MS. After 2 weeks of 5 Hz rTMS at 110% of the RMT applied to the motor cortex of patients with multiple sclerosis, the patients’ maximum voluntary contraction strength significantly increased compared to the sham stimulation group (Zanette et al., 2008). Similar results have been observed in patients with spinal cord injuries. After four consecutive weeks of rTMS treatment at 20 Hz and 110% of the RMT, with a total of 1,800 pulses per session applied to the primary motor cortex in patients with spinal cord injury, the maximum voluntary contraction of the knee flexors and extensors was increased compared to patients in the control group (Krogh et al., 2022). Sarcopenia can lead to a decrease in the conduction velocity of motor and sensory nerves, with the amplitude of compound nerve action potentials showing also a decline (Blasco et al., 2020). Evidence that rTMS can improve nerve conduction velocity was provided by Yan et al. (2023), who employed 10 Hz rTMS at 80% of the RMT, delivering a total of 1,400 pulses to the bilateral primary motor cortices of patients with multiple myeloma, an hematological disease commonly associated with sarcopenia. Following a 6 weeks intervention, rTMS increased the motor and sensory conduction velocities of the bilateral median nerve, posterior tibial nerve, ulnar nerve, and peroneal nerve in these patients. Therefore, rTMS may enhance muscle function by counteracting the reduction in nerve conduction velocity associated with sarcopenia. Moreover, rTMS might serve as an effective intervention for alleviating emotional and psychological disturbances in sarcopenia patients. The prevalence of depression in sarcopenia patients is estimated at 28% (Li et al., 2022). RTMS has been proven effective for treating depression and is approved by the United States Food and Drug Administration (FDA) (Cappon et al., 2022). Recent studies demonstrate that applying rTMS to the left dorsolateral prefrontal cortex in elderly patients with depression, at 10 Hz and 80%–110% of the RMT for 8 weeks, significantly reduces Hamilton Depression Rating Scale (HAMD) scores and effectively alleviates depressive symptoms (Wang X. et al., 2023).

In addition, numerous studies in recent years have confirmed that rTMS can improve motor disorders associated with various diseases. The latest evidence-based guidelines for rTMS treatment recommend high-frequency rTMS targeting the bilateral primary motor cortex to alleviate motor impairments in Parkinson’s disease (PD) (Level B evidence: probable effective) (Lefaucheur et al., 2020). Several studies have also validated the efficacy of 10 Hz,100% RMT rTMS applied to the primary motor cortex, revealing significant improvements in motor function among patients with PD, with effects being sustained for 30 days after treatment conclusion (Brys et al., 2016; Xie et al., 2024). Studies have also found that rTMS can enhance motor abilities and alleviate neuroinflammatory responses in in PD mice. After a 10-day rTMS intervention, PD model mice showed increased midbrain expression of anti-inflammatory mediators (IL-10 and TGF-β1), decreased expression of inflammatory cytokines (IL-6 and TNF-α), and microglial inactivation (Xie et al., 2024). In MS patients, rTMS at a frequency of 20 Hz and an intensity of 80% of the RMT, with a total of 1,200 stimuli per session, can alleviate spasticity and fatigue by modulating the excitability of the motor cortex, thereby improving motor dysfunction (Korzhova et al., 2019). The potential mechanism may be related to rTMS inducing the regeneration of myelin sheaths in demyelinated axons by increasing the number of oligodendrocyte precursor cells and promoting their differentiation (Cullen et al., 2019). Moreover, evidence indicates that rTMS may also improve motor function by enhancing neuronal plasticity in the motor cortex. Wang et al. (2021) investigated the effects of rTMS combined with treadmill training on the motor function of rats with spinal cord injury. After an 8 weeks continuous intervention, the combined treatment group showed the best recovery of motor function. Additionally, there was a significant upregulation of synaptic plasticity markers such as BDNF, PSD-95, and SYN in both the spinal cord and motor cortex, indicating that the combined therapy modulated adaptive neural plasticity at different levels of the motor system. Specifically, it was suggested that rTMS and treadmill training may selectively stimulate plasticity in the motor cortex and the spinal cord, respectively. In summary, several animal and human studies suggest that rTMS can alleviate muscle atrophy, enhance muscle strength, improve nerve conduction velocity, and enhance motor function by reducing oxidative stress and chronic inflammation and improving neural plasticity. Furthermore, the combination of rTMS with exercise therapy may yield synergistic effects. RTMS stimulation of the primary motor cortex not only modulates its excitability but also induces remote excitation of motor neurons in the corticospinal tract, influencing muscle excitability and strength (Siebner et al., 2022). Thus, the primary motor cortex appears to be a promising target for improving muscle strength in sarcopenia patients. However, no studies have yet directly applied rTMS to sarcopenia treatment. As research continues to advance, rTMS may thus become a new option for the treatment of sarcopenia, offering renewed hope for improving the quality of life in the elderly population.

## 7 Summary and outlook

As the global population ages, sarcopenia and CI have become significant factors affecting the health of the elderly. In recent years, an increasing number of scholars have recognized the interaction between these two conditions, suggesting that they mutually promote one another, further deteriorating the health status of older adults. However, effective treatments for sarcopenia and CI are currently lacking. Existing research indicates that both conditions may share similar pathophysiological mechanisms, highlighting the need to explore new therapeutic approaches for jointly treating these pathological processes. rTMS, as an innovative treatment method, has shown promise in enhancing learning, memory, and motor abilities by alleviating chronic inflammation, oxidative stress, and promoting the secretion of neurotrophic factors, demonstrating considerable potential in the management of cognitive and motor disorders (Figure 2). Therefore, rTMS may represent a new strategy for managing sarcopenia and CI, and future studies could explore its efficacy in patients suffering from both conditions. Although the above findings are encouraging, rTMS still faces several challenges in practical application. Firstly, rTMS parameters vary significantly across studies, with no consensus on optimal parameters for disease treatment, including stimulation frequency, intensity, target site, and duration. Additionally, changes in brain tissue during aging must be considered to optimize rTMS parameters. Future studies should employ techniques such as functional magnetic resonance imaging or PET-CT to precisely locate stimulation targets and tailor treatment parameters to individual patient needs, thereby achieving personalized and precise treatment. Additionally, existing studies on rTMS for treating CI often involve small sample sizes and short follow-up periods. The lack of large-scale, multicenter, and long-term clinical trial data thus precludes a comprehensive assessment of rTMS efficacy in treating CI. Therefore, it is necessary to conduct multicenter, large-sample, long-term follow-up studies in the future (e.g., 5 years cohort studies). In the context of sarcopenia treatment, although numerous studies have confirmed the potential of rTMS to enhance muscle quality, function, and alleviate depression, no research has yet specifically applied rTMS to the treatment of sarcopenia. Moving forward, we anticipate achieving more breakthroughs in rTMS research for sarcopenia, thereby offering novel insights and strategies for its comprehensive management. It is important to note that rTMS, as an adjunctive therapy, can only slow disease progression and cannot address underlying systemic issues. Thus, future research should investigate the synergistic effects of combining rTMS with exercise training, nutritional interventions, and pharmacological treatments. Research should evolve from animal model validation and Phase I safety trials to Phase III multicenter randomized controlled trials to advance the clinical application of rTMS. Although numerous scientific and clinical challenges still need to be addressed, the existing research findings and the growing interest in this area instill confidence for future investigations. From fundamental research to practical applications, rTMS is expected to become an important means to enhance quality of life for the elderly, offering new solutions to tackle the challenges of an aging society.
